# “Common sense is hard work” but benefits from persistent collaboration: Lessons learnt from the development of The Collaborative Network for European Clinical Trials for Children (c4c) to support the conduct of paediatric clinical trials of medicines

**DOI:** 10.1016/j.conctc.2025.101442

**Published:** 2025-02-03

**Authors:** Sabah Attar, Carla Peacock, Mandy Wan, Erin Halil, Chloe Bickerstaff, Lionel Tan, Hafsah Bhatti, Ricardo M. Fernandes, Regis Hankard, Mark A. Turner

**Affiliations:** a University of Liverpool, Department of Women's and Children's Health, Liverpool Women's NHS Foundation Trust, Crown Street, Liverpool, L8 7SS, UK; b Conect4children Stichting, Utrecht, Netherlands; c Evelina Pharmacy, Guys' & St Thomas' NHS Foundation Trust, London, SE1 7EH, UK; d Institute of Pharmaceutical Science, King's College London, London, SE1 9NH, UK; e Johnson & Johnson, 50-100 Holmers Farm Way, High Wycombe, HP12 4DP, UK; f ViiV Healthcare, 980 Great West Road Brentford, Middlesex, TW8 9GS, UK; g Leeds Beckett University, Leeds, LS1 3HE, England, UK; h Laboratory of Clinical Pharmacology and Therapeutics, Faculty of Medicine, University of Lisbon, Av. Prof. Egas Moniz MB, 1649-028, Lisboa, Portugal; i French Clinical Research Infrastructure Network (F-CRIN)-PEDSTART, INSERM, University of Tours, University Hospital of Orléans, Orléans, France

**Keywords:** Paediatrics, Clinical networks, Network development, Lessons learnt, Collaboration, Infrastructure

## Abstract

**Introduction:**

The Collaborative Network for European Clinical Trials for Children (c4c) is a public private partnership with a developed infrastructure across European sites to support the design and conduct of multi-national academic and industry paediatric clinical trials. This paper aims to review the learning points identified during co-development of c4c processes by academic and industry partners.

**Methods:**

Study metrics were recorded. Learning points were captured during network development, categorized and included in a thematic analysis from which lessons learnt were identified.

**Results:**

12 trials were supported by sites coordinated at national level and integrated at European level. A total of 9 CDA cycles were completed, resulting in 436 site CDAs signed in a median of 8.11 days. Lessons learnt included the importance of: relationship building by early engagement with partners; reducing misunderstanding by clear communication; flexibility, adaptability and experiential learning which are required for service improvement. Practical actions that infrastructure developers and users can take include operational planning with a view to fostering collaborations across stakeholders, sharing information about different approaches to clinical operations, and raising awareness of the need for explicit work on collaboration, communication, and planning. Traditionally, these activities are repeated for each trial. The use of a persistent network allows the benefits of collaboration to be recycled.

**Discussion:**

Building a successful framework for collaboration allows dedication and determination to carry over from one study to another. The initial investment of time to share assumptions and “state the obvious” by each user will support future trials.

## Highlights

• Categorisation of lessons learnt during set up of academic and industry trials.
• Learning from paediatric trial set-up in European network infrastructure.
• Network development within a public-private partnership.

## Abbreviations as they appear in the paper

c4c: The Collaborative Network for European Clinical Trials for Children
PoV: proof-of-viability trials
NIO: Network Infrastructure Office
NHs: National Hubs
CDA: Confidentiality Disclosure Agreement
CRO: Clinical Research Organization
IMP: Investigational Medicinal Product
PNT: Paediatric Trials Network

## 1 Background

Children were historically overlooked in drug development research, resulting in new drugs not being available or being used routinely without sufficient safety and efficacy data in this population [1]. This prompted a number of legislative initiatives, the most notable being the regulatory frameworks created in the US and Europe [2]. Although, the Paediatric Regulation came into effect in 2007 in the European Union, aiming to stimulate research and development of medicines for use in children [3], the 10-year report on the implementation of the Paediatric Regulation identified some common challenges to the conduct of paediatric clinical trials, noting in particular long set up time and delays in trial completion [4]. These challenges occur across clinical specialities [5,6] in industry and academic trials, many of which can be ascribed to a research infrastructure that is limited and not fit for purpose to address these challenges [7].

A public private partnership was established under the Innovative Health Initiative (formerly the Innovative Medicine Initiative) to address these barriers. The Collaborative Network for European Clinical Trials for Children (c4c) aims to address some of these challenges by building a network for national coordination and support of paediatric research across Europe [8]. The c4c consortium is comprised of 10 large pharmaceutical companies, 37 non-industry partners including academia, hospitals, third-sector organizations, and patient advocacy groups. The aim of c4c is to support the conduct of academic and industry sponsored paediatric trials across the trial lifecycle. 9 industry and 3 academic proof-of-viability (PoV) trials have been supported by c4c to date.

Their roll out across 21 countries is supporting the development of the network infrastructure, enabling evaluation of c4c and the developing services.

Published literature on the development of paediatric clinical research networks, or clinical research networks in general, is largely descriptive [[9], [10], [11]], with limited attention given to the underpinning stakeholder expectations and assumptions, and how these shape service development and improvement. Some clinical research networks have reported their lessons learnt during their development and early establishment, either with respect to experiences related to the set-up of networks across one or more countries [12,13], infrastructure development [[14], [15], [16]], capacity building [17], implementation [18], communications [19] or to outline the challenges and possible solutions for interoperability across networks [20]. However, there is limited literature on working within the unique dynamic of a public-private clinical research network, nor are there consolidated actions that can be undertaken to optimise the impact of clinical research networks/infrastructures.

This paper uses qualitative methods to describe how the c4c network can mitigate challenges arising during clinical trials through the deployment of a large, multinational, infrastructure that has been defined by both industry and academia.

We initially provide some context describing the scope of the c4c infrastructure and services before addressing the specific objectives of this paper.

1. Describe the services that were co-developed between industry and academia including a summary of how the services were used
2. Identify themes arising from the lessons learnt and map identified themes to the services developed
3. Identify practical implications of the lessons learnt

## 2 Context

In response to needs expressed by academia and industry, c4c developed a range of services including expert advice from subject matter experts on clinical and methodological questions and also patient and public involvement [21], multistakeholder meetings [22], education and training [23] and input to the development of paediatric data standards [24]. This paper specifically relates to c4c trial services, co-designed by industry and academic partners that were structured to support all stages of a clinical trial with coordination via an explicit governance model. These services provide strategic support and a point of reference for trial teams but do not involve any Transfer of Regulatory Obligations to c4c. The viability of the network was assessed using so-called Proof of Viability (PoV) trials that tested the services developed by the consortium: 3 academic trials that were funded by the consortium and 5 industry trials. An additional 4 industry trials were adopted by the network during the c4c project.

The Network Infrastructure Office (NIO) coordinates clinical work across 20 National Hubs (NHs) (mostly academic-based institutions with strong links to the national paediatric research and clinical networks), 21 European countries and around 220 clinical sites. The service has been transferred into a Dutch non-profit, conect4children Stichting (https://conect4children.eu)

When themes or mapping were not clear, consensus was sought between the two NIO members classifying the learning points (CP and SA), if consensus was not possible, the opinion of the senior investigator (MAT) was included.

This study involved administrative data only and so did not require review by an Ethics Committee.

## 3 Results

For a full list of learning point references and an example of learning points (n = 138) is available from the authors.

## 4 Trial support services

**Fig. 1:**
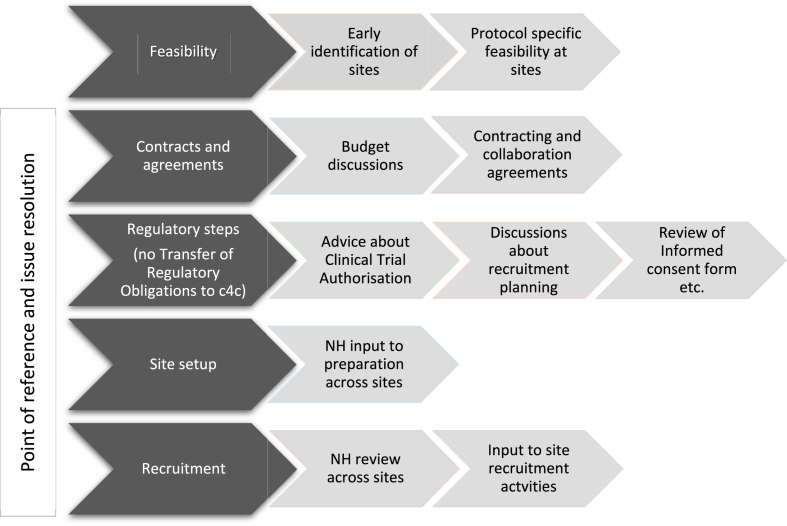
Trial support services offered by c4c (NH – National Hub).

The services are described in Fig. 1. The trials supported by the network during the project are summarised in Table 1. The use of the services is summarised in Table 2.

**Table 1:** Summary of 9 c4c Proof of Viability and supplementary studies and services delivered.

Industry Sponsor	c4c Trial identifier	Therapeutic area	Services delivered
Sponsor 1	I-PoV1	Multiple Sclerosis	All c4c services
Sponsor 2	I-PoV2	Chronic Kidney Disease	All c4c services
Sponsor 3	I-PoV4	Respiratory Syncytial Virus	All c4c services
Sponsor 4	I-PoV3	Asthma	All c4c services
Sponsor 3	I-PoV5	Ulcerative Colitis	All c4c services
Sponsor 4	I-Adn1	Hypercholesterolemia	Site Identification and Feasibility
Sponsor 4	I-Adn2	Adolescent Asthma	Site Identification
Sponsor 2	I-Adn3	Chronic Kidney Disease	Recruitment Support
Sponsor 4	I-Adn4	Hypercholesterolemia	Site Identification and Feasibility Site Set-up

**Table 2:** Summary of the CDA and feasibility metrics for the 9 c4c Proof of Viability and supplementary studies.

c4c Trial identifier	Number of NHs involved	Number of NH-Site CDAs signed	Median NH-Site CDA	Number of Feasibilities Completed	Median NH-Site TSQ	Number of selected sites
I PoV1	8	43	4.75	10	13	19
I PoV2	19	108	5.13	86	9	48
I PoV3	18	88	5.08	72	10.65	13
I PoV4	13	46	15.91	23	26.04	19
I PoV5	12	68	8.11	56	14.93	27
I Adn1	9	6	23.96	4	41.29	0
I Adn2	3	15	8.28	12	17.25	NA
I Adn3	16	39	11.03	NA	NA	41
I Adn4	10	23	4.03	18	14.92	tbc

At the time of writing another 11 studies have been discussed with the network and are at various stages of support. 5 companies have placed, or plan to place, more than one study.

## 5 Themes

**Fig. 2:**
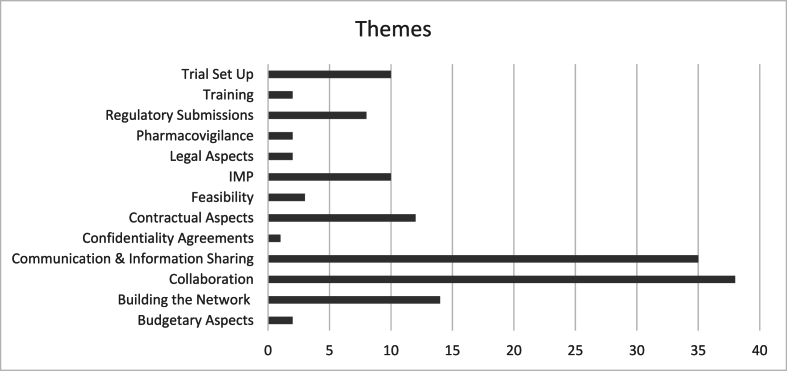
Number of points related to Themes.

The themes used for classification of the learning points are shown in Fig. 2. Perhaps unsurprisingly, based on the nature of the infrastructure that was developed, by far the most learning points were related to “Collaboration” and “Communication and information sharing”. Whereas far fewer learning points were flagged in the categories of “Confidentiality” and “Legal Aspects”.

## 6 General lessons

Following the review of all the themes and their contributing learning points, six lessons have been extracted.

### 6.1 Lesson 1: early engagement with stakeholders builds relationships

By reaching out to trial Sponsors at the earliest opportunity, c4c was able to develop open communication pathways facilitating regular discussions. This enabled the setting of clear expectations and escalation processes and proved to be a solid foundation to building open and resilient relationships, tailoring the pathways to meet the needs of the sponsor. Early engagement enabled c4c to understand Sponsor processes and ways of working and adapt services to harmonize with Sponsor activity and develop trusting partnerships. Everyone assumed this would be easy but intensive discussions were valuable.

### 6.2 Lesson 2: clear, simple and efficient communication across sites and sponsors promotes understanding

Gathering clear expectations from the Sponsor was critical in ensuring accurate deployment of services to avoid duplication of effort at sites, frustration by investigators and sponsor team, and potential inadvertent relationship issues. Sponsor organizations are complex, so this needed time, energy, and commitment. However, the investment in a stable system for strategic communication will pay off across multiple trials.

Communications across a multinational infrastructure needed to be clear, simple, concise, avoid informal idioms, timely and proactive, and to encourage feedback from all parties involved with a view to learn from and to improve the divergent infrastructure. For example, the provision of clear clinical trial requirements and sponsor expectations from the outset enabled thorough high-quality feasibility from NHs and sites and consequently, more informed stakeholders. These communications take significant effort: good communication does not happen by accident.

### 6.3 Lesson 3: knowledge of site and country specific requirements assist in addressing local challenges

Understanding of country requirements in terms of regulatory submissions, contract agreements and country specific governing laws was useful. For example, some countries require clinical trial agreements in place before ethics/competent authority submissions. Awareness of different approaches to standard of care, that reflect national culture and regimes, means that protocol flexibility within the constraints of the protocol is required. Sites have specific reasons for their internal processes reflecting the issues they have encountered in the past. Changing internal site processes is not easy, particularly if different trials require different modifications to how sites work. We have developed a novel point of reference for reconciliation between Sponsors and sites using multiple national hubs coordinated at the European level.

### 6.4 Lesson 4: experiential learning coupled with flexibility and adaptability support service improvement within and between trials

The advantages of “learning from doing” naturally incorporated a continuous cycle of reflection and improvement to develop efficient services, including the ability to adjust to meet the needs and requirements of each stakeholder and situation.

A “one size fits all approach” is too rigid and doesn't work when engaging with multiple sites across multiple countries. Thus, flexibility was required, whilst striving to streamline and standardize processes for maximum efficiency. The learnings from most trials are lost. Our approach allows sites and Sponsors access to lessons learnt without having to “reinvent the wheel” for each trial.

### 6.5 Lesson 5: fostering collaboration between partners is key to success

A cascading confidentiality disclosure agreement (CDA) was introduced to expedite this key step in trial feasibility. This approach was initially met with resistance, with some trial sites requesting changes to CDA clauses; however, following substantial collaboration, the CDA templates were approved.

Positive Sponsor feedback reported that the cascading CDA process was extremely efficient, allowing local teams to focus on other trial related tasks, and is indicative of this successful collaboration.

By continually educating Sponsor and CRO teams on the purpose of c4c and services, knowledge base, experience and support available from c4c NHs to progress trial activities, ensures that c4c will be recognized as a supporting collaborator to complement resources already available.

### 6.6 Lesson 6: planning and mitigation for clinical operations

Thorough planning and mitigation related to logistics is critical to proactively prevent potential problems and delays during set up and execution of a clinical trial. For industry trials, all aspects of sourcing and distribution of the Investigational Medicinal Product (IMP) are managed and coordinated centrally by the trial sponsor; however, for academic trials, where the responsibility may be placed on sites, it is easy to assume consistency across countries and sites which, as we have learnt, is not the case. A list of IMP considerations is provided in Appendix A.

## 7 Implementation

The network has taken the learnings and implemented changes to activities and processes to improve our services, some of the completed actions are described below.

· Developed and implemented a NH Profile to help identify differences across NHs in terms of country/site specific requirements, regulatory processes, clinical practice
· Updated c4c customer facing guidance, brochures to better explain what c4c is, the benefits and value in collaboration
· Improved initial contact processes to improve communication by setting expectation, encouraging transparency and collaboration.
· Developed and executed a RASCI process and templates to establish clear roles and responsibilities across all stakeholders
· Created a Case Study catalogue from experiences so far which can be utilized to show how c4c can collaborate in trial delivery bringing benefits and value to the sponsor

A recommendation for the future sustainable network is to retain the approach of regularly tabling “lessons learnt” on meeting agendas and maintaining a catalogue to capture lessons ensuring continuous improvement and implementation of actions.

## 8 Discussion

We have described the services developed and lessons learnt from the establishment of a public-private clinical research network to support pediatric clinical trials across Europe. Common learning points across the themes such as a proactive approach, early engagement, collaboration, communication, education, and awareness are comparable to the experiences of other clinical research networks/infrastructures (referenced in the introduction).

The importance of effective communication was a recommendation across the various themes (***Lesson 2***) being consistent with the findings of GRACE1 whilst establishing a primary care network across 13 European countries [13] (Nuttal et al.). This reinforces our approach to the development of a network that addresses pediatric specificities. All clinical trials involve a lot of work to align sponsor, CROs if relevant, and sites. Individuals may assume that alignment is easy, but experience suggests that alignment takes time and effort. We have described an approach that allows this effort to be reused across multiple trials, although an initial investment of time and effort in the network is needed by each user.

IMP has been noted by other pediatric clinical trial experiences as a factor that impedes trial set up [[25], [26], [27]] (***Lesson 6***). Within c4c, a systemic solution was piloted by integrating expertise in IMP supply and use from the early stage of trial planning. Potential IMP challenges and solutions were identified and raised at the trial feasibility stage via the c4c IMP advisory service enabling sponsors to integrate workable IMP solutions into the trial design and operational plan. Nonetheless, some IMP problems encountered during trial set up could have been addressed earlier along with local national input, emphasizing the need for ongoing proactive management [28]. Pharmacovigilance solutions implemented through a coordinated service utilizing the NHs was one approach used by c4c that has proven to be effective [29,30].

“Learning from doing” (***Lesson 4***); success in this area required regular consultation across the c4c network. We have applied to pediatrics the experience of other networks who have developed a continuous feedback loop to ensure iterative improvements [12] (Powers et al.) taking account of the unique specificities of research at pediatric sites.

We found that initial expectations of designing the processes began to unravel during the implementation of the processes. For collaborative work, expectations that sound similar during preliminary discussions can turn out to be divergent when actual work is done. Users and providers were expected to use shared processes and information. Information sharing is not always undertaken and over-communication in early stages of collaboration is more beneficial (***Lesson 1, Lesson 5***). For processes, extensive preparatory work is needed. The more complex and heterogenous an organization and/or its customer base is, the more use cases are required to develop a service that is ‘fit for purpose’. c4c provides an ideal context for these discussions because the work will feed forward into future research.

An expectation that organizations are relatively homogenous and that within companies, trial teams and national affiliates, all follow the same processes was premature. There are multiple levels of complexity across pharmaceutical companies, academic research institutions and hospitals. Siloes are to be expected and time is needed to develop useful discussions between people from different siloes. We speculate that these complexities are one barrier to successful research and that a paediatric-specific competence network can mitigate the recurrent challenges that arise from divergent sponsor cultures.

With respect to consistency in countries, it was found that the drivers for internal consistency within hospitals and institutions are much stronger than drivers to collaboration (**Lesson 3**). Ongoing country-level activities, background knowledge and relationships of the National Hubs facilitate navigating this local landscape across stakeholders and partners, including both sites and local sponsor or CRO teams. The US Paediatric Trials Network (PTN) experience of setting up a similar network to study paediatric off patent drugs is consistent with what is outlined in this paper, although PTN works in a single, non-European country [31].

As a result of the lessons learnt, the network has achieved a number of key innovations.

· Improved the speed and efficiency of the CDA cascade process.
· Highlighted the value of the NHs, one example being their ability to resolve issues between sites and Sponsors.
· Tested and proved the validity of the c4c services developed during the project, generating ready to use services for the sustainable network.
· Demonstrated the added value of the c4c network by creating a suite of supporting case studies.
· Developed National Hub profiles which assist in identifying country requirements and variations across countries.

## 9 Limitations of the study

The c4c project commenced in 2018 with the initial 12–18 months utilized for the development of the services. The time window of review of lessons learnt, Jan 2021–May 2022, may not have captured important aspects of set-up of network services that occurred before this time. Additionally, as the project progresses through delivery of the PoV trials and more trials are added to the c4c portfolio, there will be additional lessons learned going forward.

Relevance and generalizability of some of the lessons, particularly with respect to delays at clinical sites, need to be reviewed in the context of the COVID-19 pandemic that occurred during the set-up phase [32,33].

## 10 Conclusion

Building a successful collaboration for one or multiple trials requires dedication and determination. While the lessons described in this paper may appear obvious, there is often an assumption in collaborations that stakeholders work in similar ways. However, as discussed, this isn't the case and can lead to misunderstanding and unanticipated challenges. To resolve these challenges, collaborators, whether from large or small institutions, must be receptive to listening to and learning from the approaches taken by others and be willing to pivot and adapt ways of working to improve processes. C4c provides a framework for alignment that is informed by deep understanding f pediatric specificities during the design and conduct of clinical trials that recruit babies, children and young people. The commonsense approach requires effort for each new user and the network but enables the stakeholders in the collaboration to move forward to achieve their shared vision.

## CRediT authorship contribution statement

**Sabah Attar:** Writing – review & editing, Writing – original draft, Supervision, Project administration, Methodology, Investigation, Formal analysis, Data curation, Conceptualization. **Carla Peacock:** Writing – review & editing, Writing – original draft, Visualization, Supervision, Methodology, Investigation, Formal analysis, Data curation, Conceptualization. **Mandy Wan:** Writing – review & editing, Writing – original draft, Validation. **Erin Halil:** Writing – review & editing, Validation. **Chloe Bickerstaff:** Formal analysis, Data curation. **Lionel Tan:** Writing – review & editing, Validation. **Hafsah Bhatti:** Writing – original draft. **Ricardo M. Fernandes:** Writing – review & editing, Validation. **Regis Hankard:** Writing – review & editing, Validation. **Mark A. Turner:** Writing – review & editing, Writing – original draft, Supervision, Project administration.

## Disclaimer

The publication reflects the author's view and neither IMI nor the European Union, EFPIA, or any Associated Partners are responsible for any use that may be made of the information contained therein.

## Declaration of generative AI in scientific writing

The use of AI tools has not been used to analyse and draw insights from data as part of the research process.

## Funding statement

The conect4children (c4c)-Collaborative Network for European Clinical Trials for Children project has received funding from the Innovative Medicines Initiative 2 Joint Undertaking under grant agreement No 777389. The Joint Undertaking receives support from the 10.13039/501100007601European Union's Horizon 2020 research and innovation programme and 10.13039/100013322EFPIA.

## Declaration of competing interest

The authors declare the following financial interests/personal relationships which may be considered as potential competing interests: Lionel Tan is an employee of ViiV Healthcare and holds shares in GSK. Erin Halil is an employee of J&J and holds shared in the company. Sabah Attar, Carla Peacock, Mandy Wan, Hafsah Bhatti, Ricardo M Fernandes, Regis Hankard, Mark A. Turner declare that they have no known competing financial interests or personal relationships that could have appeared to influence the work reported in this paper.

## Data Availability

Data will be made available on request.
