# Supplementary material for: “Common sense is hard work” but benefits from persistent collaboration: Lessons learnt from the development of The Collaborative Network for European Clinical Trials for Children (c4c) to support the conduct of paediatric clinical trials of medicines
Source: Contemp Clin Trials Commun. 2025 Feb 3;44:101442. doi: 10.1016/j.conctc.2025.101442 (PMC11850778; doi:10.1016/j.conctc.2025.101442)
Supplement: Multimedia component 1 [file mmc1.docx]

Appendix A.

Table A.1 lists IMP considerations from c4c Non-Industry Proof of Viability trials

| The drugs evaluated in the 4 academic trials were established medicinal products for which there are limited pediatric data for the conditions under study. While these trials did not necessitate new pharmaceutical product development, the importance of integrating expertise in IMP supply and use from the early stage of trial planning was noted. The key learning points were: | |
| --- | --- |
| 1 | Decisions about IMP supply must include a comprehensive assessment of the pharmaceutical characteristics of these products to determine their suitability for use in children, along with considerations for the specific trial design. |
| 2 | Decision-making regarding IMP supply is intrinsically linked to trial design and can greatly influence both the operational delivery strategy and budget of a trial. |
| 3 | Access to authorized medicinal products differs across EU countries, and there is a need for greater transparency to allow for thorough product evaluations that can guide decision-making regarding IMP supply. |
| 4 | Trials that rely on sourcing established medicinal products from the open market are susceptible to supply interruptions that are beyond the sponsor's control. In some instances, alternative approaches like direct sourcing from the manufacturer might prove advantageous. |
| 5 | IMP plans should include contingencies to address potential delays in starting the trial or slow participant recruitment, along with the associated costs and time needed to replace expired IMP. |
| 6 | Differences in what is regarded as standard care for drug treatment, such as dosing, medicine preparation, and administration, can compromise the integrity of a trial. Engaging with sites during the planning phase allows for early conversations about protocol standardization, while also accommodating necessary flexibility. |
| 7 | Having access to experts with country-specific knowledge of medicine supply, trial regulatory requirements, and pharmacy practice can enhance communication and problem-solving between sponsors and site teams, thereby facilitating more timely trial set up. |

Table A.1: IMP considerations
